# Impact of socioeconomic factors and health determinants on preterm birth in Brazil: a register-based study

**DOI:** 10.1186/s12884-022-05201-0

**Published:** 2022-11-24

**Authors:** Luciano de Andrade, Arthi S. Kozhumam, Thiago Augusto Hernandes Rocha, Dante Grapiuna de Almeida, Núbia Cristina da Silva, Rejane Christine de Souza Queiroz, Miyoko Massago, Sharla Rent, Luiz Augusto Facchini, Antônio Augusto Moura da Silva, Catherine Ann Staton, João Ricardo Nickenig Vissoci, Erika Barbara Abreu Fonseca Thomaz

**Affiliations:** 1 Department of Medicine, State University of Maringa, Block 126, Colombo Avenue, 5790, Parana CEP: 87020-900 Maringa, Brazil; 2 Duke Global Health Institute, Duke University, Durham, NC USA; 3 Federal University of Minas Gerais, Belo Horizonte, Minas Gerais Brazil; 4 Department of Public Health, Federal University of Maranhão, São Luís, Maranhão Brazil; 5 Department of Social Medicine, Federal University of Pelotas, Pelotas, Rio Grande Do Sul Brazil

**Keywords:** Brazil, Preterm birth, Child health, Maternal health, Primary health care or Primary health care

## Abstract

**Background:**

More than 15 million children are born preterm annually. While preterm survival rates have increased in high-income countries. Low- and middle-income countries, like Brazil, continue to battle high neonatal mortality rates due to a lack of adequate postnatal care. Globally, neonatal mortality is higher for preterm infants compared to those born at term. Our study aims to map and analyze the spatial, socioeconomic, and health coverage determinants related to preterm birth in Brazil in order to understand how spatial variations in demographics and access to primary care may affect preterm birth occurrences.

**Methods:**

Using publicly available national-level data from the Brazilian health system for 2008–2017, we conducted an ecological study to visualize the spatial distributions of preterm birth along with socioeconomic status, the structure of health services, and primary care work process, each consisting of multiple variables reduced via principal component analysis. Regression models were created to determine predictive effects of numeric and spatial variation of these scores on preterm birth rates.

**Results:**

In Brazil, preterm birth rates increased from 2008–2017, with small and rural municipalities frequently exhibiting higher rates than urban areas. Scores in socioeconomic status and work process were significant predictors of preterm birth rates, without taking into account spatial adjustment, with more positive scores in socioeconomic status predicting higher preterm birth rates (coefficient 0.001145) and higher scores in work process predicting lower preterm birth rates (coefficient -0.002416). Geographically weighted regression showed socioeconomic status to be a more significant predictor in the North, with the work process indicators being most significant in the Northeast.

**Conclusions:**

Results support that primary care work process indicators are more significant in estimating preterm birth rates than physical structures available for care. These results emphasize the importance of ensuring the presence of the minimum human resources needed, especially in the most deprived areas of Brazil. The association between social determinants of health and preterm birth rates raises questions regarding the importance of policies dedicated to foster equity in the accessibility of healthcare services, and improve income as protective proxies for preterm birth.

**Supplementary Information:**

The online version contains supplementary material available at 10.1186/s12884-022-05201-0.

## Background

More than 15 million children are born preterm, defined as birth prior to 37 completed weeks of gestation [1, 2], each year. Though preterm survival rates have increased in high-income countries, low- and middle-income countries (LMICs) like Brazil still see high rates of neonatal mortality that is often attributed to a lack of adequate postnatal care [3]. Preterm birth (PTB) in LMICs is a growing global public health concern, and among other causes is associated with a lower quality of prenatal preventative care [4]. Brazil ranks among countries with the highest number of PTB [2]. Data from the National Live Births System (SINASC) in Brazil showed that in 2017 the rate of PTB was 11 per 100 live births [5], a higher rate than other Latin American and Caribbean countries [3].

Regional differences in health outcomes in Brazil are a well-known issue, including those regarding the surgical workforce for children [6], microcephaly and Zika virus [7], and cardiac diagnostic testing [8]. PTB appears to show similar spatial variability: while overall rates of PTB in Brazil increased from 6.5% in 2004 to 10.9% in 2017, the distribution of PTB was not balanced across the five official regions: North, Northeast, Center-West, Southeast, and South [9, 10].

To reduce regional disparities in healthcare, Brazil has advanced the implementation of a Universal Health Coverage (UHC) program, the SUS (Sistema Único de Saúde). Created in 1990, the SUS is the national public health system and provides decentralized UHC services through networks of clinics and healthcare facilities [11]. The Brazilian SUS and social security structures have several information systems that systematically collect data from the population in areas including healthcare utilization, healthcare quality, and social vulnerability. As part of SUS, the Family Health Strategy is a community-based program providing primary health care (PHC) services through Family Health Teams composed of at least family physicians, nurses, nursing technicians, and community health agents [12]. Evidence suggests that improvements in PHC resources in Brazil have the potential to reduce PTB rates [13], and that UHC can improve life expectancy at birth [14] and ensure healthcare services required to children’s growth [15].

Health conditions (e.g., maternal diabetes, pregnancy history), sociodemographic factors (e.g., low education and socioeconomic status), health habits (e.g., stress and work habits) and health care infrastructure (e.g., quality of care, number of prenatal care visits) are well-known predictors of PTB worldwide [10, 16–23]. Regional inequalities exist in Brazil in regards to socioeconomic status [24], healthcare services [25], and healthy life expectancy [26]. Several studies have analyzed factors related to PTB in Brazil including socio-demographics and availability of prenatal care [27–29], however none have utilized a geographic information systems (GIS) approach to geospatially map the rates and locations of these factors in association with PTB.

GIS approaches have been shown to be a promising research strategy to understand social and geographic determinants of health [30, 31]. Analyzing the effect of predictors over the spatial distributions of PTB may be a useful approach to uncover spatially-dependent risk and protective factors for PTB in Brazil. The use of GIS-based approaches may generate insights capable of improving maternal and child health across the municipalities. There is a lack of studies using sophisticated geospatial modeling techniques, such as geographically weighted regression (GWR), to better understand the spatial PTB risk, as well as the potential impact of socio-demographic determinants on prematurity. Our study aims to map and analyze the spatial, socioeconomic, and the health coverage determinants related to PTB in Brazil in order to understand how spatial variations in demographics and access to quality primary care may affect PTB occurrences.

## Methods

### Study design

We conducted a longitudinal, ecological study based on regularly collected Brazil health system data following the Strengthening the Reporting of Observational Studies in Epidemiology (STROBE) protocol [32]. The data analyzed regarding PTB in Brazil ranged from 2008 through 2017. The following variables were considered: PTB rates, socioeconomic data, physical structure of primary care facilities, and healthcare work process. The structure and work process of health services were initially collected by each health facility. Socioeconomic data was collected at the municipality level. The overall unit of analysis was at municipality level.

### Study population, data sources and variables assessed

Three groups of variables were considered to estimate PTB risk: data regarding PTB characteristics; data concerning the quality of primary care, measured through the availability of physical structure to offer care, as well as the work process performed by the primary care teams; and social determinants of health, considered as confounders to explain the municipality-level PTB rates. Combining data from multiple health information systems with social determinants of health data, we examined the impact of socio-demographics on PTB rates in Brazil using a geospatial approach.

The Brazilian Ministry of Health (MoH), by Live Births Information System (SINASC), is responsible for sharing all information collected regarding the epidemiological information of live birth outcomes in the country [5, 33]. We used this system to retrieve data about week of birth, prenatal care, newborn weight, Apgar score, mother's age at birth date, municipality of birth, and type of delivery rate of PTB from 2008 to 2017.

The National Program for Improving Primary Care Access and Quality (PMAQ) is a monitoring survey conducted biannually to evaluate the quality of the health care teams [34]. This survey provided data on infrastructure of health services as well as PHC work processes in Brazil. Within the PMAQ, the “structure of health services” refers to static structures and “work process” represents dynamic activities, among the activities included in this Program there are the territorialization and sanitary accountability, accessibility, reception and preferential gateway, longitudinal care, comprehensive network care management, and so on. The PMAQ was based on Donabedian’s structure—process—outcome model, with embedded strategies for qualification, monitoring, and evaluation of health teams that is linked to a financial incentive for municipalities that meet set standards of access and quality [35]. The program is organized into biennial cycles developed in continuous and complementary phases [36]. According to the Brazil MoH, PMAQ data consisted of 17,202 teams across 3,944 municipalities (in 2012–2013) and 29,778 teams across 5,040 municipalities (in 2013–2014). The data corresponding to the first (2012) and second (2014) PMAQ cycles (https://aps.saude.gov.br/ape/pmaq) were considered in the analysis of this manuscript.

Data for social determinants came from the 2010 census in Brazil, most recent information available. To assess the impact of these indicators in the PTB rates, we evaluated data regarding average life expectancy (years), rate of under 1 year old mortality by 1,000 live birth, rate of under 5 year old mortality by 1,000 live birth, % of the population in the municipality without formal education, % of the population in the municipality with 8 or less years of education, GINI index of the municipality (a synthetic indicator that captures the level of inequality for a given variable and population), % of the population in the municipality categorized as living in poverty, and average per capita income within municipality.

To evaluate the aforementioned predictors affecting the PTB rates, we used data from 29,178,429 live births distributed across the 5,562 Brazilian municipalities, between the years of 2007 and 2017. The raw indicators analyzed were grouped according to the structure described in the supplementary material.

### Data analysis-analytical approach

#### Spatial distribution and autocorrelation

In order to analyze the predictive relationships between PTB and health coverage determinants, we first aimed to understand PTB distribution in Brazil. We mapped the average spatial distributions of PTB rates (number of PTB/100 births) across two time periods, 2008–2012 and 2013–2017, to reduce possible nonspecific and irregular variations which can lead to misunderstandings of the results. We performed spatial autocorrelation to visualize clusters of PTB for both time periods across Brazil municipalities. Spatial autocorrelation measures the presence of dispersion or clustering in a region through Moran’s I (Index). The Moran's I index assumes a positive or negative value varying between -1 and 1, with a value of zero representing the hypothesis of spatial interdependence [37]. However, since a negative global Moran’s I value may not necessarily indicate the absence of spatial correlation on a local level [38], local indicators of spatial association (LISA) were applied by calculating and evaluating the significance of each city’s Moran’s I at alpha = 0.05, to find de clusters with high rates of PTB. The clusters are visualized on choropleth maps and characterized as follows: high-high (a city with a high PTB rate is close to others that also exhibit high PTB rates), high-low (a city with a high PTB rate is adjacent to others that have low PTB rates), low–high (a city with a low PTB rate is adjacent to others that have high PTB rates), low-low (cities with low PTB rates adjacent to others that also have low PTB rates) and NS (no significance, in which cities with high PTB rates are surrounded by cities with both high and low PTB rates).

#### Predictive modeling

We built a GWR model, aggregating data from socioeconomic status, structure of health services, and primary care work process seeking to forecast the PTB rate for each Brazilian municipality. To assess the spatial effect of social determinants on PTB rates, we performed an additional analytical step split into two approaches. The first analytical approach aimed to reduce the dimensionality of the predictors through principal component analysis (PCA) to meet the statistical requirements of GWR analysis. This first step generated factors summarizing the raw indicators selected for the analysis. These resulting factors were used as proxies of the predictors concerning socioeconomic status and the structure and work process of primary care services. The second analytical step used the GWR model to estimate the levels of PTB rates in the Brazilian municipalities. The predictors considered for GWR analysis came from the factors resulting from dimensionality reduction.

1. First analytical step- PCA for dimensionality reduction.

Since each domain (socioeconomic status, primary care work process, and structure of health services) consists of multiple variables, it is important to avoid using variables that are highly correlated due to multicollinearity issues. The use of several variables with overlapping variance can artificially inflate the portion of variance explained by each, thus creating a validity problem. The main strategy to overcome this is using dimensionality reduction approaches. Therefore, we used PCA on the indicators concerning socioeconomic status, structure of health services, and primary care work process to obtain a smaller, non-overlapping, number of factors representing each of the predictors. PCA is an orthogonal linear transformation [39] such that the largest eigenvalues (principal components) can be used to reconstruct a large amount of the variance of the original data [40]. The appropriate number of variables to retain for each domain was determined using component loadings and Scree plots (created to display total variation in each domain explained by each component). One factor was retained for each one of the three dimensions considered in the present study. The three factors were considered as predictors to generate a spatially weighted regression model aiming to forecast the PTB rate by municipality.

2. Second analytical step- GWR models considering the factors representing health determinants and their impact on PTB rates.

To understand associations between PTB and factors from the three health coverage domains, we adopted an approach based on spatially weighted regression models. The spatial dependency highlights the situations in which the local of the occurrence of a phenomenon can be at least partially explained by the geospatial perspective. To verify if the observed behavior regarding the PTB distribution over the Brazilian territory is modulated by the spatial dimension, we first ran a regression model using the Ordinary Least Square (OLS) approach without weighting the model for the spatial dimension. The OLS regression model minimizes the sum of square differences between predicted and observed values, leading to model parameter estimates without geographic representation [41].

The residuals of the regression model represent the magnitude of the distance between the forecasted values and the actual ones. Coefficients of the model must be statistically significant, without heteroscedasticity, and residuals normally distributed without spatial autocorrelation [42]. If the residuals of the OLS model are spatially correlated, this suggests the presence of spatial dependency of the object being studied. Thus, residuals from the initial OLS model were analyzed for spatial self-correlation using Moran’s I to evaluate the amount of PTB that could be explained by the spatial perspective. Once spatial dependency is observed, the best suitable model to forecast the variation in PTB rates is a GWR model. Since the residuals from the OLS predictive PTB analysis showed a strong spatial self-correlation, we used GWR to identify possible local associations and demonstrate the spatial effect of the multivariate model. GWR is a spatial analysis technique that takes non-stationary variables like environmental characteristics into account, to model predictive relationships [43].

GWR models use multivariate regression to evaluate associations between potential predictors and an outcome measure, weighting the results by the locality of occurrence [44]. The GWR model tested the PTB rate by municipality as the outcome measure, and the PCA factors regarding the socioeconomic status, structure of health services, and primary care work process as predictors. All analyses were performed using RStudio [45]. PCA was conducted using the *psych* package in R. The spatial self-correlation and OLS model were processed using GeoDa software 1.10.0.8 (Spatial Analysis Laboratory, Urbana, IL) [46], and the GWR model by GWR 4.0 [47]. Choropleth maps were created using QGIS 2.14 software [48].

## Results

### Spatial distribution of PTB and spatial autocorrelation

**Fig. 1:**
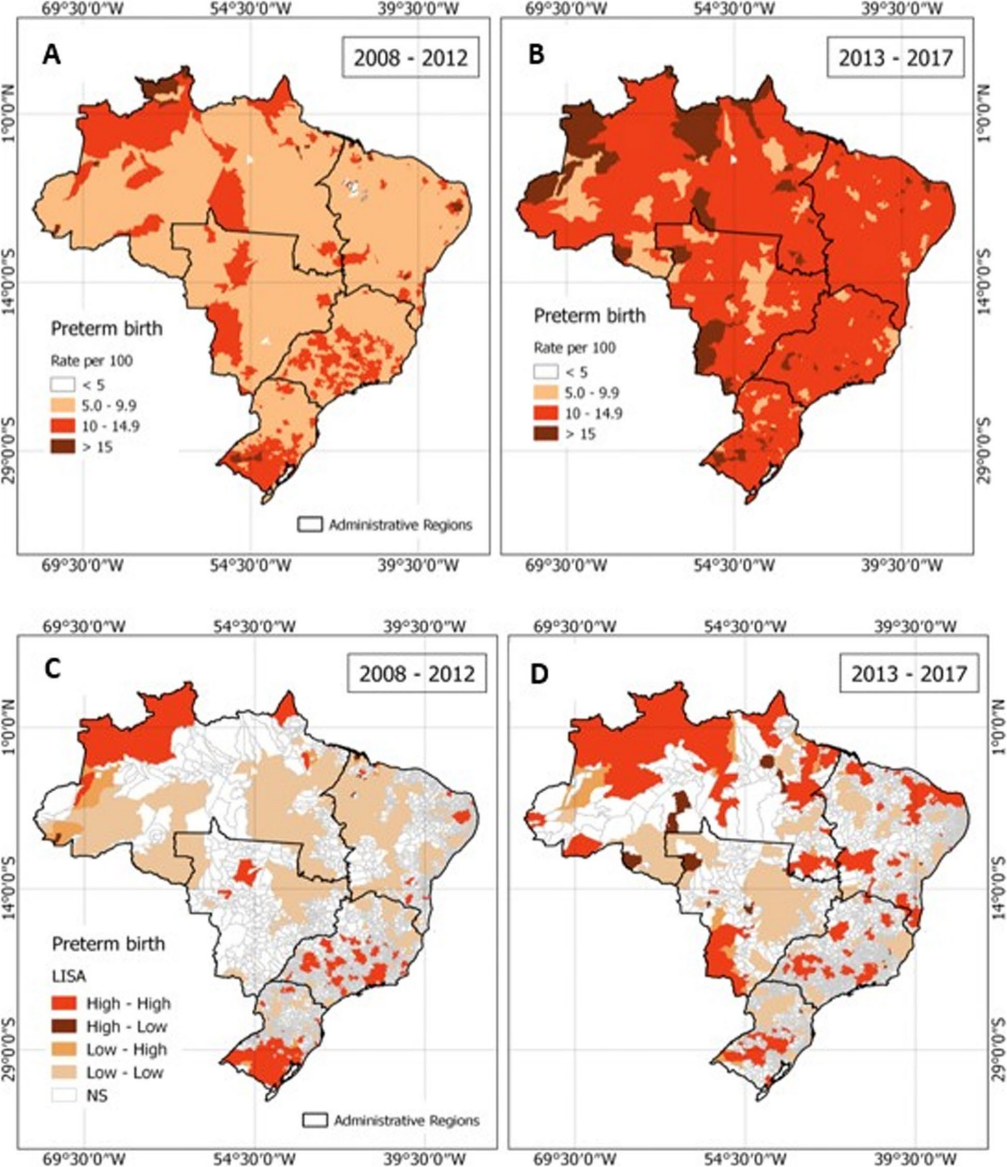
Distribution of preterm birth rate (per 100 births) by Brazilian municipalities (top) and spatial autocorrelation (bottom) across two-time frames. The plot of each time frame reflects an average rate over that time period. Data were extracted from the National Register of Live Births in Brazil via the SINASC from 2008–2017- Autocorrelation was conducted via the local indicators for spatial association (LISA) spatial cluster analysis, measuring spatial association within adjacent cities within each administrative region

Between the first (2008–2012) and second (2013–2017) ranges of years, we saw an increase in PTB rate across the majority of municipalities nationwide. From 2008 to 2012, PTB was highest in the North, South and Southeast portion of the country. Between 2013 and 2017, PTB rates were at least 5–9.9% across all cities, with more cities in the North reporting rates higher than 15%. During the 2013–2017 time period, nearly all regions of the country reported an increase in their PTB rate (Fig. 1A, B).

In the 2013–2017 time period, the analysis of spatial correlation of the raw PTB rates, when compared to the 2008–2012 results, showed a larger number of high-high clusters and fewer low-low clusters (Fig. 1C,D). We observed fewer areas of “no significance” in the second time period (especially in the North portion of the country), indicating more similar PTB rates between adjacent municipalities. The number of municipalities categorized as a high-high cluster reflects an increasing trend in terms of PTB occurrence in the second period analyzed.

### PCA dimensionality reduction and GWR analysis

The PCA loads attributed to the variables regarding socioeconomic status, structure of health services, and primary care work process are significantly different across the five regions of Brazil. (Tables 1, 2 and 3). The loadings coefficients represent a linear combination predicting a variable by the (standardized) components. Higher loadings coefficients identify which variables have the largest effect on each component resulting from the PCA analysis. Loadings close to -1 or 1 indicate that the variable strongly influences the component. A positive value represents a direct association between the variable considered and the principal component representing the dimension reduced, and the negative value represents the opposite.

**Table 1:** Descriptive analysis and PCA component loadings of socioeconomic status variables

**Social determinants of health indicators**		**Brazilian Regions**					**ANOVA across regions** F value, Pr(> F)	**PCA component loading**
	**Brazil** Median (IQR)	**South** Median (IQR)	**Southeast** Median (IQR)	**Midwest** Median (IQR)	**Northeast** Median (IQR)	**North** Median (IQR)		
Average life expectancy (years)	73.47 (71.15–75.16)	75.15 (74.03–76.16)	74.72 (73.55–75.84)	74.34(73.71–75.03)	70.44 (74.03–76.16)	71.76 (70.60–73.04)	2319 < 0.001	-0.918
Rate of under 1 year old mortality by 1,000 live births	16.90 (13.80–23.80)	12.80 (11.50–14.40)	15.40 (13.57–17.10)	15.17 (13.80–17.40)	26.30 (23.00–30.70)	21.20 (18.00–24.60)	2873 < 0.001	0.908
Rate of under 5-year-old mortality by 1,000 live births	19.42 (16.04–25.82)	14.99 (13.37–16.80)	17.82 (15.69–19.85)	18.15 (16.32–21.02)	28.36 (24.83–33.32)	22.70 (19.28–26.44)	2200 < 0.001	0.900
Percentage of municipality without formal education	12.90 (7.70–23.80)	7.15 (4.6–10.40)	9.00 (6.40–13.00)	11.70 (9.13–14.20)	26.90 (22.80–31.20)	16.30 (12.40–20.90)	2576 < 0.001	0.914
Percentage of municipality with 8 or less years of education	39.09 (32.35–46.74)	43.16 (37.76–48.90)	44.48 (36.91–51.70)	42.70 (39.06–47.40)	31.67 (27.76–36.80)	36.74 (31.26–42.68)	37,381 < 0.001	-0.810
GINI index of the municipality	0.54 (0.49–0.61)	0.49 (0.45–0.54)	0.53 (0.49–0.64)	0.56 (0.51–0.60)	0.56 (0.52–0.60)	0.59 (0.56–0.63)	135,292 < 0.003	0.483
Percentage of municipality categorized as living in poverty	25.11 (18.62–41.91)	15.44 (10.22–23.44)	20.55 (17.78–25.64)	21.06 (18.32–29.89)	57.07 (41.70–67.83)	48.36 (37.37–66.72)	1,636,680 < 0.003	0.944
Average per capita income within municipality	752.13 (502.04–1109.01)	858.00 (706.50–1108.10)	904.80 (657.60–1416.10)	821.70 (629.20–1305.40)	359.40 (258.83–584.31)	475.60 (307.10–738.40)	459,165 < 0.001	0.872
Percentage of households in municipality covered with water and sanitation supply	99.39 (99.20–99.63)	99.40 (99.16–99.61)	99.30 (99.24–99.61)	99.11 (98.04–99.45)	99.56 (99.22–99.72)	99.44 (99.18–99.55)	10,393 < 0.001	0.720
HDI index of the municipality	0.67 (0.60–0.72)	0.72 (0.69–0.74)	0.71 (0.66–0.74)	0.69 (0.67–0.71)	0.59 (0.56–0.61)	0.61 (0.57–0.65)	1794 < 0.001	-0.966

Label: *PCA* Principal component analysis, *IQR* interquatile range, *HDI* Human development index

**Table 2:** Descriptive analysis and PCA component loadings of structure of health services variables

**Structure of health services average values (2012–2014)**		**Brazilian Regions**					**ANOVA across regions** F value,Pr(> F)	**PCA component loading**
	**Brazil** Median (IQR)	**South** Median (IQR)	**Southeast** Median (IQR)	**Midwest** Median (IQR)	**Northeast** Median (IQR)	**North** Median (IQR)		
Percentage of primary care teams within the municipality that schedule referrals to specialized health consultation	95.00 (52.85–100.00)	97.50 (60.00–100.00)	97.75 (95.45–100.00)	62.50 (40.00–93.94)	60.00 (15.87–88.24)	66.67 (20.00–93.33)	205,505 < 0.001	0.454
Percentage of primary care teams using health information systems	100.00 (96–100)	97.50 (60.00–100.00)	97.75 (95.45–100.00)	62.5 (40.00–93.94)	66.67 (20.00–93.33)	60.00 (15.87–88.24)	18,488 < 0.001	0.585
Percentage of primary care teams within the municipality that register the data of pregnant women receiving health care consultations	99.35 (94.74–100.00)	90.90 (89.19–100.00)	99.12 (97.06–100.00)	98.68 (94.45–100.00)	100.00 (94.44–100.00)	100.00 (96.43–100.00)	9654 < 0.001	0.595
Percentage of primary care teams within the municipality that register the data of patients receiving dental consultations	70.63 (40.00–85.77)	75.00 (42.86–95.00)	71.43 (45.65–85.77)	50.00 (32.14–85.14)	60.86 (43.75–80.56)	57.14 (33.33–80.00)	14,851 < 0.001	0.494
Percentage of primary care team within the municipality that deliver exams to pregnant women in a timely manner to conduct necessary interventions	83.33 (56.25–94.94)	91.39 (72.73–100.00)	88.91 (71.43–95.44)	81.82 (42.86–96.97)	60.00 (40.00–78.12)	66.67 (40.00–87.50)	160,169 < 0.001	0.537
Percentage of primary care teams within the municipality that utilize penicillin	76.99 (20.00–99.61)	97.95 (5000–100.00)	98.48 (21.74–99.61)	73.68 (41.67–100.00)	36.36 (11.11–75.00)	71.43 (40.62–100.00)	85,978 < 0.001	0.489
Percentage of primary care teams within the municipality that offers a referral for ultrasound exam	86.14 (60.71–100.00)	88.89 (68.97–100.00)	81.62 (47.47–98.93)	66.67 (52.73–97.30)	90.91 (75.00–100.00)	85.71 (66.67–100.00)	46,202 < 0.001	0.569
Average number of physicians per primary care center within a municipality	1.22 (1.00–2.20)	1.50 (1.00–2.33)	1.30 (1.00–3.00)	1.30 (1.00–2.33)	1.00 (1.00–1.50)	1.28 (1.00–2.00)	66,928 < 0.001	0.549
Average number of nurses per primary care center within a municipality	1.50 (1.00–3.00)	1.40 (1.00–2.21)	2 (1–3.13)	1.44 (1.00–3.00)	1.00 (1.00–1.73)	1.38 (1.00–2.00)	109,957 < 0.001	0.634
Average number of dentists per primary care center within a municipality	1.00 (0.89–2.00)	1.00 (0.95–1.50)	1.47 (0.92–2.33)	1.00 (1.00–2.00)	1.00 (0.86–1.25)	1.00 (0.83–1.33)	53,457 < 0.001	0.594
Percentage of primary care teams in the municipality offering health care services during two shifts a day	100.00 (99.00–100.00)	100.00 (100.00–100.00)	100(99.51–100)	100.00 (94.55–100.00)	100.00 (97.56–100.00)	100.00 (99.34–100.00)	12,272 < 0.001	0.604
Average number of medical consultation clinics per primary care team within the municipality	100.00 (100.00–100.00)	100.00 (100.00–100.00)	100(99.9–100)	100.00 (99.09–100.00)	100.00 (100.00–100.00)	100.00 (98.68–100.00)	2839 < 0.001	0.415
Average of dental consultation clinics per primary care team within a municipality	97.53 (68.18–100.00)	98.36 (70.59–100.00)	95.45(69.23–100)	100.00 (68.18–100.00)	93.90 (73.08–100.00)	91.67 (50.00–100.00)	7190 < 0.001	0.298
Percentage of municipality’s primary care centers with medical records for pregnant women	100.00 (81.25–100.00)	100.00 (98.41–100.00)	96.3(73.58–100)	100.00 (83.33–100.00)	100.00 (75.00–100.00)	100.00 (85.71–100.00)	34,025 < 0.001	0.645
Percentage of municipality’s primary care centers with tetanus/diphtheria vaccine always available	94.59 (50.00–100.00)	100.00 (66.67–100.00)	94.59(53.7–100)	90.00 (57.14–100.00)	94.12 (50.00–100.00)	83.33 (44.74–100.00)	3981 < 0.001	0.719
Percentage of municipality’s primary care centers with influenza vaccine always available	96.00 (50.00–100.00)	85.71 (33.33–100.00)	99.12(50–100)	80.00 (40.00–100.00)	94.29 (50.00–100.00)	90.00 (45.39–100.00)	7796 < 0.001	0.710
Percentage of municipality’s primary care centers with Hepatitis B vaccine always available	100.00 (70.27–100.00)	100.00 (66.67–100.00)	100(72.41–100)	95.24 (58.18–100.00)	100.00 (77.78–100.00)	95.92 (50.00–100.00)	9516 < 0.001	0.713
Percentage of municipality’s primary care centers with at least one of each of certain equipment materials (at least one scale, glucometer, sonar, clinical table, spotlight for gynecological examination)	99.61 (95.95–100.00)	100.00 (96.88–100.00)	100.00 (98.37–100.00)	99.66 (95.68–100.00)	98.05 (95.24–100.00)	95.95 (87.50–100.00)	47,694 < 0.001	0.623
Percentage of municipality’s primary care centers with at least one of each of the materials listed (at least measuring tape, speculum, endocervical brush, ayres spatula)	98.29 (90.00–100.00)	100.00 (90.00–100.00)	98.29 (92.5–100.00)	97.57 (87.27–100.00)	96.67 (90.00–100.00)	85.13 (80.00–100.00)	74,674 < 0.001	0.612
Percentage of municipality’s primary care centers with sufficient availability of mineral salts, vitamin B9 and ferrous sulfate	85.71 (57.78–100.00)	88.24 (66.67–100.00)	95.83 (62.86–100.00)	69.70 (37.50–100.00)	82.33 (64.29–100.00)	66.67 (36.23–83.33)	18,275 < 0.001	0.502

Label: *PCA* Principal component analysis, *IQR* interquatile range

**Table 3:** Descriptive analysis of structure of work process variables

**Work process average values (2012–2014)**		**Brazilian Regions**					**ANOVA across regions** F value, Pr(> F)	**PCA component loadings**
	**Brazil** Median (IQR)	**South** Median (IQR)	**Southeast** Median (IQR)	**Midwest** Median (IQR)	**Northeast** Median (IQR)	**North** Median (IQR)		
Percentage of primary care teams within the municipality offering prenatal care consultations	91.18 (62.50–100)	88.24 (66.67–100.00)	97.64 (81.40–100.00)	75.00 (54.00–96.97)	77.41 (51.35–100.00)	60.00 (48.48–93.88)	62,141 < 0.001	0.420
Percentage of primary care teams within the municipality with ability to provide referral for any medical exams	97.06 (90.62–100.00)	100.00 (94.44–100.00)	97.06 (95.51–100.00)	93.94 (82.14–100.00)	96.59 (83.33–100.00)	87.50 (50.00–98.33)	122,599 < 0.001	0.530
Percentage of primary care teams within the municipality that register pregnant medical data	100.00 (99.41–100.00)	100.00 (100.00–100.00)	100.00 (99.41–100.00)	100.00 (98.65–100.00)	100.00 (97.56–100.00)	100.00 (100.00–100.00)	3012 < 0.001	0.773
Percentage of primary care teams within the municipality that register vaccination of pregnant women’s medical data	100.00 (95.24–100.00)	100.00 (94.74–100.00)	98.73 (97.06–100.00)	97.30 (93.75–100.00)	100.00 (95.82–100.00)	100.00 (93.42–100.00)	5536 < 0.001	0.670
Percentage of primary care teams within the municipality that register cytopathological exams of pregnant women’s medical data	89.19 (70.00–97.95)	91.78 (75.00–100.00)	94.78 (75.00–96.00)g	85.45 (75.00–100.00)	83.33 (66.67–92.86)	71.71 (58.33–89.19)	25,065 < 0.001	0.647
Percentage of primary care teams within the municipality that offer guidance regarding the tetanus vaccine to pregnant women	100.00 (100.00–100.00)	100.00 (100.00–100.00)	100.00 (99.62–100.00)	100.00 (99.35–100.00)	100.00 (100.00–100.00)	100.00 (100.00–100.00)	6772 < 0.001	0.826
Percentage of primary care teams within the municipality that offer medical consultations	100.00 (100.00–100.00)	100.00 (100.00–100.00)	100.00 (99.90–100.00)	100.00 (99.09–100.00)	100.00 (100.00–100.00)	100.00 (98.68–100.00)	2839 < 0.001	0.745
Percentage of primary care teams within the municipality that offer nursing consultations	100.00 (100.00–100.00)	100.00 (100.00–100.00)	100.00 (99.18–100.00)	100.00 (100.00–100.00)	100.00 (100.00–100.00)	100.00 (100.00–100.00)	51,057 < 0.001	0.812
Percentage of primary care teams within the municipality that offer dental consultations	97.53 (68.15–100.00)	98.36 (70.59–100.00)	95.45 (69.23–100.00)	100.00 (68.18–100.00)	93.90 (73.08–100.00)	91.67 (50.00–100.00)	7190 < 0.001	0.533
Percentage of primary care teams within the municipality that offer drugs to pregnant women	100.00 (89.09–100.00)	100.00 (91.67–100.00)	100.00 (87.51–100.00)	100.00 (75.00–100.00)	100.00 (97.30–100.00)	100.00 (72.46–100.00)	8154 < 0.001	0.565
Percentage of primary care teams within the municipality that offer vaccinations to pregnant women	100.00 (66.67–100.00)	100.00 (77.78–100.00)	99.71 (50.00–100.00)	100.00 (70.00–100.00)	100.00 (94.12–100.00)	100.00 (51.97–100.00)	41,030 < 0.001	0.610
Percentage of primary care teams within the municipality that offer syphilis tests	0.00 (0.00–50.00)	0.00 (0.00–40.00)	3.33 (0.00–83.95)	0.00 (0.00–50.00)	0.00 (0.00–25.00)	0.00 (0.00–50.00)	27,316 < 0.001	0.335
Percentage of primary care teams within the municipality that offer pregnancy tests	0.00 (0.00–88.00)	0.00 (0.00–7.69)	75.00 (0.00–100.00)	0.00 (0.00–42.86)	0.00 (0.00–15.38)	0.00 (0.00–14.29)	205,751 < 0.001	0.281
Percentage of primary care teams within the municipality that offer HIV tests	8.73 (0.00–87.50)	10.00 (0.00–82.39)	14.29 (0.00–100.00)	0.00 (0.00–66.67)	0.00 (0.00–46.02)	7.25 (0.00–81.82)	27,676 < 0.001	0.376
Percentage of primary care centers in the municipality with sufficient availability of antihypertensive medication	92.68 (45.33–100.00)	95.12 (50.00–100.00)	96.07 (50.00–100.00)	75.00 (12.50–100.00)	85.29 (50.00–100.00)	66.67 (33.33–100.00)	14,414 < 0.001	0.491
Percentage of primary care teams within the municipality with sufficient availability of antidiabetic medication	50.00 (16.67–82.67)	50.00 (25.00–87.50)	50.00 (00.0–89.11)	50.00 (2.78–75.00)	50.00 (34.38–75)	50.00 (22.83–68.33)	2729 < 0.001	0.394
Percentage of primary care centers within the municipality with sufficient availability of antibacterial medicine	25.11 (18.62–41.91)	15.44 (10.22–23.44)	20.55 (17.78–25.64)	21.06 (18.32–29.89)	57.07 (41.70–67.83)	48.36 (37.37–66.72)	1,636,680 < 0.001	0.566

Label: *PCA* Principal component analysis, *IQR* interquatile range

Dimensionality reduction through PCA for the groups of variables described in Tables 1, 2 and 3 established the following components as most important to explain the total variance of the dimensions assessed: i. For socioeconomic status, the percentage of population living in poverty, life expectancy, HDI index, and formal education were most important; ii. Tetanus/diphtheria and hepatitis B vaccine availability were the most important for the structure of health services; and iii. Offering guidance for the tetanus vaccine, nursing consultations, and registering pregnant medical data (work process) were most important for the primary care work process.

**Fig. 2:**
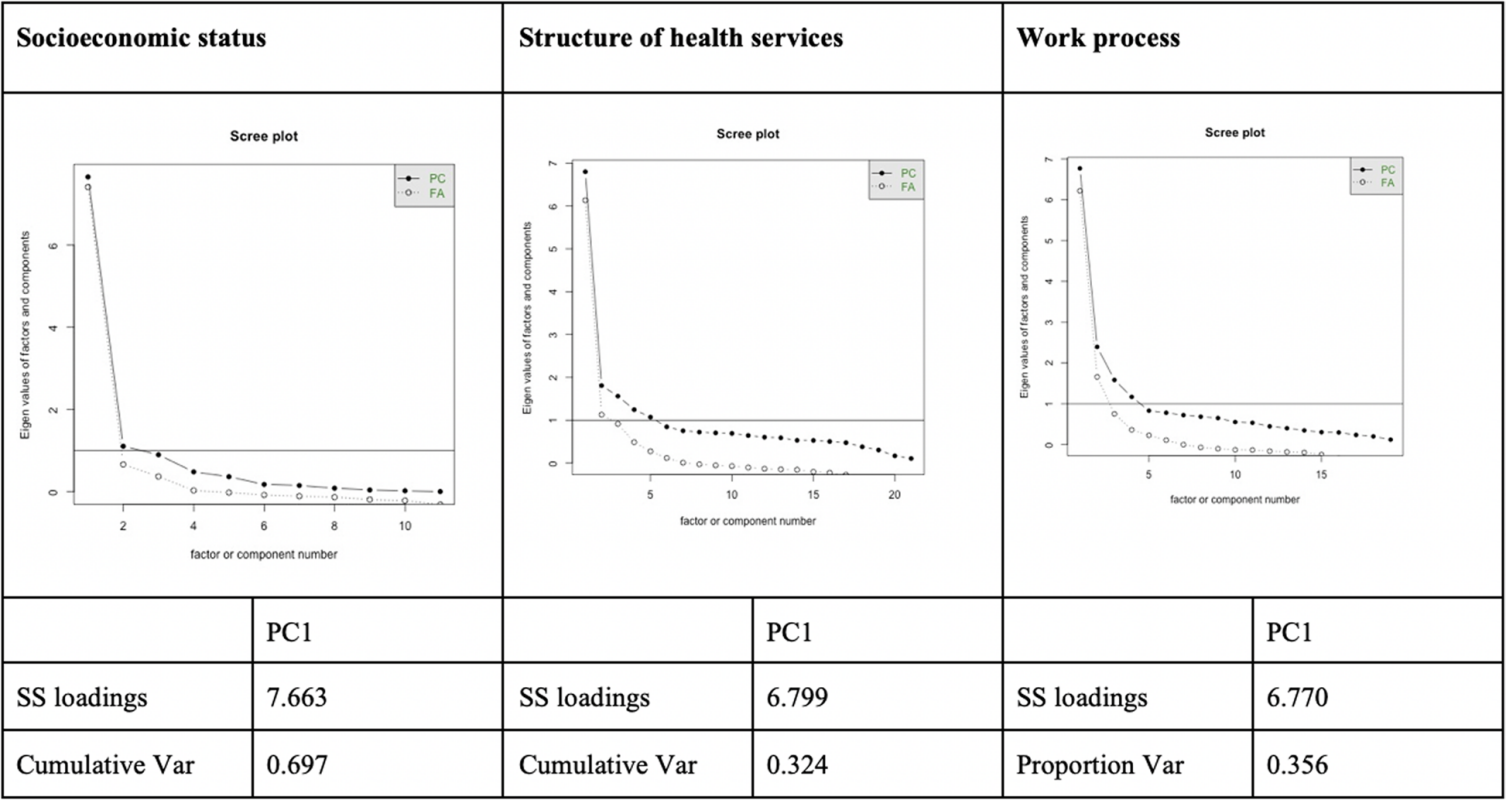
Scree plots and variance statistics across loadings for all three domains. Label: PC = principal component, FA = factor analysis, SS = sum of squares, cumulative var = cumulative variance, proportion var = proportion variance

**Fig. 3:**
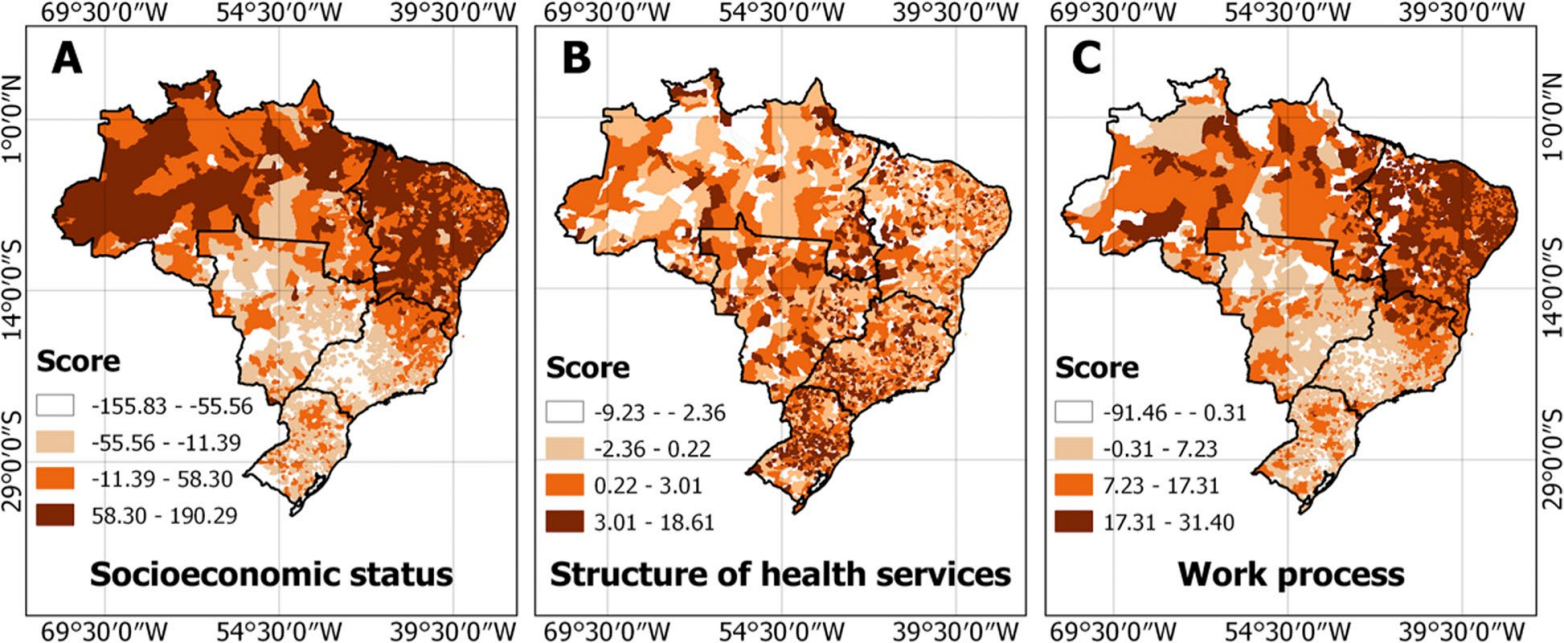
Distributions of socioeconomic status, structure of health services, and work process scores across Brazilian municipalities. Higher scores in socioeconomic status represent less positive and worse situations, while higher scores in the structure of health services and work process represent more availability of structure and work process

Scree plots showing variance of each component loadings can be examined in Fig. 2. The PCA scores regarding socioeconomic status were more positive in the North and Northeast part of the country. For the work process, positive values were observed in the Northeast region of Brazil (Fig. 3).

The factor loadings obtained were used as predictors to perform a GWR analysis. Scores in both socioeconomic status and work process were significant predictors of PTB rates, without taking into account spatial adjustment (OLS model), with socioeconomic status directly related to PTB rates (t value higher than 1.96), and maternal primary care work process was inversely related to PTB rates (t value lower than -1.96) (Table 4). By analyzing the GWR output, it was possible to observe that socioeconomic status is a more significant predictor in the North, with the work process most significant in the Northeast. The structure of health services does not appear to be a significant predictor of PTB across any region of Brazil (Fig. 3).

**Table 4:** Comparison of the OLS and GWR multivariate spatial regression models

Variable	OLS Model (Global Model)				GWR Model (Local Model)
	**Coefficient**	**SD**	**t**	**p**	
Constant	12,157	0.02153	564.57	0.00000	-
Socioeconomic status	0.001145	0.00034	3.34	0.00082	-
Structure of health services	-0.002534	0.00658	-0.38	0.70038	-
Maternal primary care work process	-0.002416	0.00096	-2.50	0.01213	-
AIC		21,059			17,345
Adjusted R2		0.003			0,580
Sum of residual squares		14,334,02			4427,55
Moran I (residual)		0,689			0,265

Label: *OLS* ordinary least squares, *GWR* Geographically weighted regression, *SD* standard deviation, *AIC* Akaike information criterion, *Moran I* Moran’s Index

**Fig. 4:**
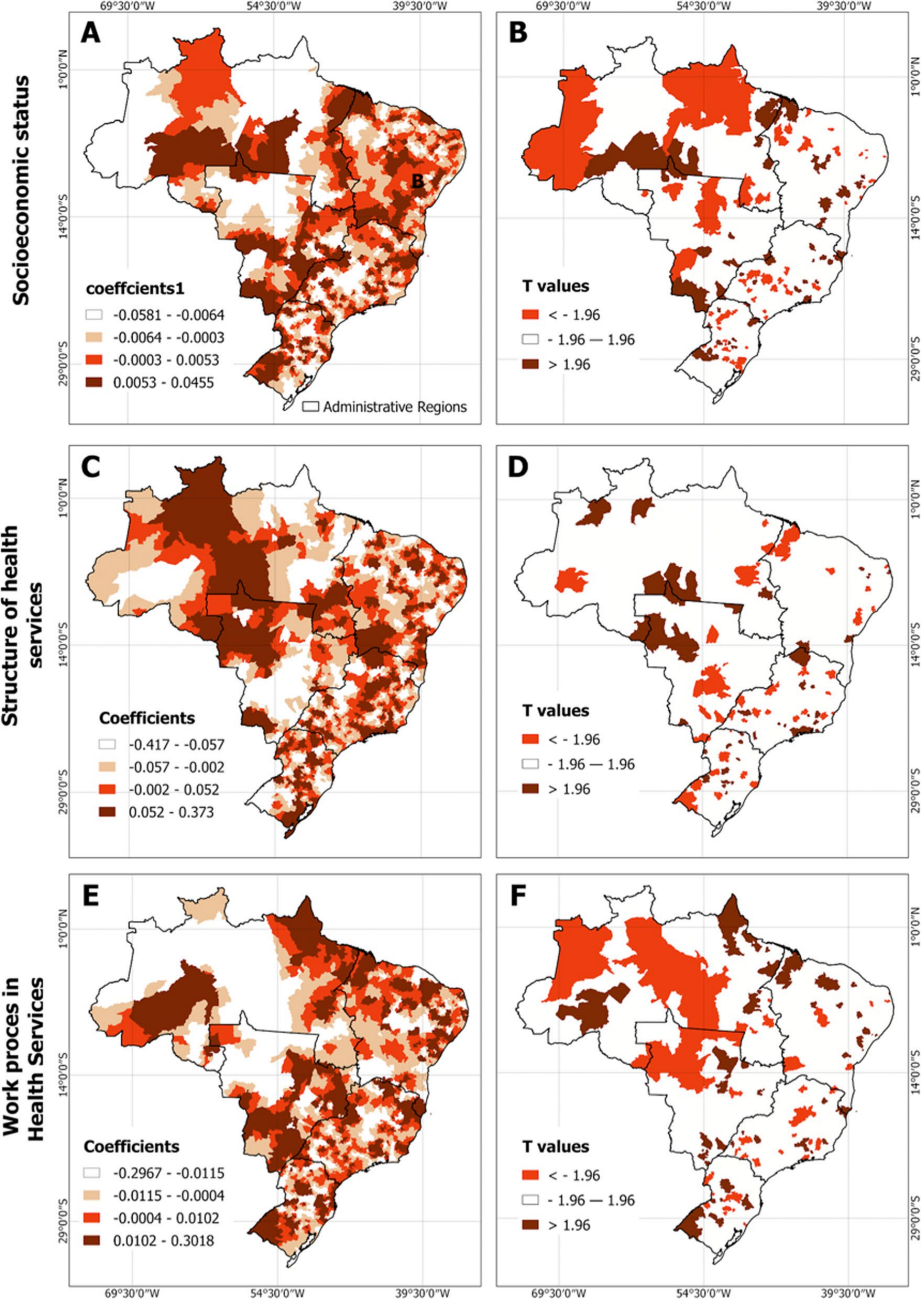
Coefficients and significance factors from GWR model of socioeconomic status, structure of health services, and work process scores predicting PTB across Brazilian municipalities

Figure 4 represents the statistical significance and the coefficients obtained by the GWR analysis. The maps on the right side reflect the municipalities in which the PCA predictors selected were significantly associated with the PTB rates and the directionality of the association. Positive values represent a trend in which an increase in the predictors is directly related to the outcome, and negative values are flagging the opposite trend. The maps on the left side are highlighting the magnitude of the observed PCA coefficients to estimate the PTB rate. The coefficients in the left side represent the intensity of association between the predictors and the outcome, and should be considered as relevant only for the significant areas flagged in the maps of the right side.

The GWR coefficients regarding the socioeconomic status were correlated negatively to PTB in the North, Midwest, and in portions of the Southeast and South regions. The orange areas highlighted as -1.96 in Fig. 4-B calls attention to municipalities presenting opposite trends between the PTB rate and socioeconomic status. The brown group of municipalities highlights areas in which an increase in the socioeconomic status is associated also with an increase in the PTB rates.

In terms of the structure of health services, shown in panel D of Fig. 4, a larger number of municipalities present opposite trends between availability of primary care physical structure and PTB rates (orange areas). Only a few municipalities registered a direct spatial association between primary care structure and the levels of PTB rates.

The analysis of the primary care work process followed the general trends observed in the other 2 domains. Large extensions of the North and Midwest regions exhibited a negative association between the work process and the PTB rates. The regions presenting scattered municipalities categorized as having a direct association between the primary care work process and the PTB levels could be observed in the North and South regions of the country.

## Discussion

The global increase in PTB rates over the past two decades is important for clinicians, researchers, and health policy makers. Understanding which factors affect PTB is an important element in understanding this phenomenon and fostering coordinated actions to tackle this challenge. Considering there is a lack of studies using a geospatial approach to characterize PTB rates, we performed a novel ecological study aiming to estimate PTB rates in Brazil using social determinants and PHC characteristics as predictors.

Our findings indicate increasing PTB rates across all regions of Brazil from 2008–2017. As PTB remains a risk factor for under-5 mortality and future morbidity [49], regional trends in PTB must continue to be monitored. Results from the GWR indicate that socioeconomic status and PHC services can be used as proxies to estimate the level of PTB rates in some regions of Brazil. The results obtained highlight the presence of regional disparities in factors positively or negatively associated with PTB. The impact of the social determinants of health and primary care quality were not the same nationwide. Our geospatial analysis highlights priority regions for combating high rates of PTB through targeted health policies and interventions.

Our findings regarding socioeconomic impact on PTB obtained in the OLS analysis are aligned with some literature on the direct relationships between maternal socio-demographics and PTB in Brazil [27, 28, 50, 51]. Our results demonstrated that primary care services availability may be important to reduce the PTB, especially in the most deprived regions, as observed in the North region of Brazil. For some municipalities, the primary care service is the only available health service within a 3- or 4-h distance. Our findings delineate existing regional disparities of birth health outcomes in Brazil [9, 10, 52], with the higher socioeconomic status in the South regions, and primary care service accessibility of the Northeast, being protective against PTB. The association between social determinants of health and PTB level raises questions regarding the importance of policies dedicated to foster equity in healthcare service accessibility, and income improvements as protective proxies for PTB.

Policy-makers and public health officials in Brazil may consider expanding existing research on health services [53–55] and exploring why certain health services are underused. Further research is also warranted into the availability and use of valuable health services, as PCA suggested eight variables (four from socioeconomic status, one from the structure of health services, and three from primary care work process) to be most reflective of the variance of each of these domains. Our study demonstrated that the primary care work process may provide a better means to estimate PTB rates, rather than the physical health system components. Indeed, the ability of health professionals to offer care is more strongly linked to reported PTB levels than is the regional distribution of equipment or supplies. These results emphasize the importance of ensuring the presence of sufficient clinical staff and human resources, especially in the most resource-limited areas of the country.

This study has a few important limitations. First, despite the advantage of using a geographically weighted regression model, the presence of other geographic barriers to healthcare access or spatial predictors, such as exposure to pollutants, may have impacted the variance of the outcome measure. Second, due to the limited availability of data regarding other factors potentially associated with PTB, our results only took into consideration some of the potential predictors associated with our outcome measure. Lastly, the use of secondary data regarding administrative records in our analysis inserts the potential for selection bias due to inadequate registering of events. To combat the risk of ecological fallacy due to the nature of this study, our analysis of data by municipality takes into account the impact of inequality at intra-state and municipal levels.

## Conclusions

Our findings support that primary care work process indicators are more significant in estimating PTB rates than physical structures available for care. These results emphasize the importance of ensuring the presence of the minimum human resources needed, especially in the most deprived areas of Brazil. The association between social determinants of health and PTB rates raises questions regarding the importance of policies dedicated to foster equity in the accessibility of healthcare services, and improve income as protective proxies for preterm birth. So, further on this topic should investigate the impact of social determinants of health at the individual level and the impact on birth outcomes.

## Supplementary Information

**Additional file 1:** **Supplementary material.** Indicators assessed to forecast PTB at the municipality level in Brazil.

## Data Availability

The data used for the present manuscript can be found at: https://figshare.com/articles/dataset/Preterm_GIS_data/21215282.

## Abbreviations

GWR: Geographically Weighted Regression
I: Index
LISA: Local Indicators for Spatial Association
LMIC: Low- and middle-income countries
MoH: Ministry of Health
OLS: Ordinary Least Square
PCA: Principal Component Analysis
PMAQ: National Program for Improving Primary Care Access and Quality
PTB: Preterm Birth
SINASC: Live Births System
STROBE: Strengthening the Reporting of Observational Studies in Epidemiology
SUS: Sistema Único de Saúde
UHC: Universal Health Coverage
