# Supplementary material for: Impact of socioeconomic factors and health determinants on preterm birth in Brazil: a register-based study
Source: BMC Pregnancy Childbirth. 2022 Nov 24;22:872. doi: 10.1186/s12884-022-05201-0 (PMC9685869; doi:10.1186/s12884-022-05201-0)
Supplement: Supplementary file 1 — Additional file 1: Supplementary material. Indicators assessed to forecast PTB at the municipality level in Brazil. [file 12884_2022_5201_MOESM1_ESM.docx]

**Supplementary material:** Indicators assessed to forecast PTB at the municipality level in Brazil.

| Domain | Indicators assessed |
| --- | --- |
| Social determinants of health indicators | Average life expectancy (years)  Rate of under 1 year old mortality by 1,000 live births  Rate of under 5-year-old mortality by 1,000 live births  Percentage of population without formal education  Percentage of population with 8 or less years of education  GINI index of the municipality  Percentage of population categorized as living in poverty  Average per capita income within municipality  Percentage of households in municipality covered with water and sanitation supply  HDI index of the municipality  Percentage of people living in low-income conditions in the municipality |
| Structure of health services | Percentage of primary care teams within the municipality that schedule referrals to specialized health consultation  Percentage of primary care teams using health information systems  Percentage of primary care teams within the municipality that register the data of pregnant women receiving health care consultations  Percentage of primary care teams within the municipality that register the data of patients receiving dental consultations  Percentage of primary care team within the municipality that deliver exams to pregnant women in a timely manner to conduct necessary interventions  Percentage of primary care teams within the municipality that utilize penicillin  Percentage of primary care teams within the municipality that offers a referral for ultrasound exam  Average number of family health teams per primary care center within a municipality  Average number of physicians per primary care center within a municipality  Average number of nurses per primary care center within a municipality  Average number of dentists per primary care center within a municipality  Percentage of primary care teams in the municipality offering health care services during two shifts a day  Average number of medical consultation clinics per primary care team within the municipality  Average of dental consultation clinics per primary care team within a municipality  Percentage of municipality’s primary care centers with medical records for pregnant women  Percentage of municipality’s primary care centers with tetanus/diphtheria vaccine always available  Percentage of municipality’s primary care centers with influenza vaccine always available  Percentage of municipality’s primary care centers with Hepatitis B vaccine always available  Percentage of municipality’s primary care centers with at least one of each of certain equipment materials (at least one scale, gliso meter, sonar, clinical table, spotlight for gynecological examination)  Percentage of municipality’s primary care centers with at least one of each of the materials listed (at least measuring tape, speculum, endocervical brush, ayres spatula)  Percentage of the municipality's primary care centers with sufficient availability of mineral salts, vitamin B9 and ferrous sulfate. |
| Primary care work process | Percentage of primary care teams within the municipality offering prenatal care consultations  Percentage of primary care teams within the municipality with ability to provide referral for any medical exams  Percentage of primary care teams within the municipality that register pregnant medical data  Percentage of primary care teams within the municipality that register vaccination of pregnant women’s medical data  Percentage of primary care teams within the municipality that register cytopathological exams of pregnant women’s medical data  Percentage of primary care teams within the municipality that offer guidance regarding the tetanus vaccine to pregnant women  Percentage of primary care teams within the municipality that offer medical consultations  Percentage of primary care teams within the municipality that offer nursing consultations  Percentage of primary care teams within the municipality that offer dental consultations  Percentage of primary care teams within the municipality that offer drugs to pregnant women  Percentage of primary care teams within the municipality that offer vaccinations to pregnant women  Percentage of primary care teams within the municipality that offer syphilis tests  Percentage of primary care teams within the municipality that offer pregnancy tests  Percentage of primary care teams within the municipality that offer HIV tests  Percentage of primary care centers in the municipality with sufficient availability of antihypertensive medication  Percentage of primary care teams within the municipality with sufficient availability of antidiabetic medication  Percentage of primary care centers within the municipality with sufficient availability of antibacterial medicine |

**Label:** HDI = Human development index
